# Differences in the alveolar macrophage toponome in humanized SP-A1 and SP-A2 transgenic mice

**DOI:** 10.1172/jci.insight.141410

**Published:** 2020-12-17

**Authors:** David S. Phelps, Vernon M. Chinchilli, Judith Weisz, Lili Yang, Debra Shearer, Xuesheng Zhang, Joanna Floros

**Affiliations:** 1 Penn State Center for Host defense, Inflammation, and Lung Disease (CHILD) Research and Departments of Pediatrics,; 2 Public Health Sciences, and; 3 Obstetrics and Gynecology, The Pennsylvania State University College of Medicine, Hershey, Pennsylvania, USA.

**Keywords:** Immunology, Pulmonology, Innate immunity, Macrophages, Pulmonary surfactants

## Abstract

Alveolar macrophages (AMs) are differentially regulated by human surfactant protein-A1 (SP-A1) or SP-A2. However, AMs are very heterogeneous and differences are difficult to characterize in intact cells. Using the Toponome Imaging System (TIS), an imaging technique that uses sequential immunostaining to identify patterns of biomarker expression or combinatorial molecular phenotypes (CMPs), we studied individual single cells and identified subgroups of AMs (*n* = 168) from SP-A–KO mice and mice expressing either SP-A1 or SP-A2. The effects, as shown by CMPs, of SP-A1 and SP-A2 on AMs were significant and differed. SP-A1 AMs were the most diverse and shared the fewest CMPs with KO and SP-A2. Clustering analysis of each group showed 3 clusters where the CMP-based phenotype was distinct in each cluster. Moreover, a clustering analysis of all 168 AMs revealed 10 clusters, many dominated by 1 group. Some CMP overlap among groups was observed with SP-A2 AMs sharing the most CMPs and SP-A1 AMs the fewest. The CMP-based patterns identified here provide a basis for understanding not only AMs’ diversity, but also most importantly, the molecular basis for the diversity of functional differences in mouse models where the impact of genetics of innate immune molecules on AMs has been studied.

## Graphical abstract

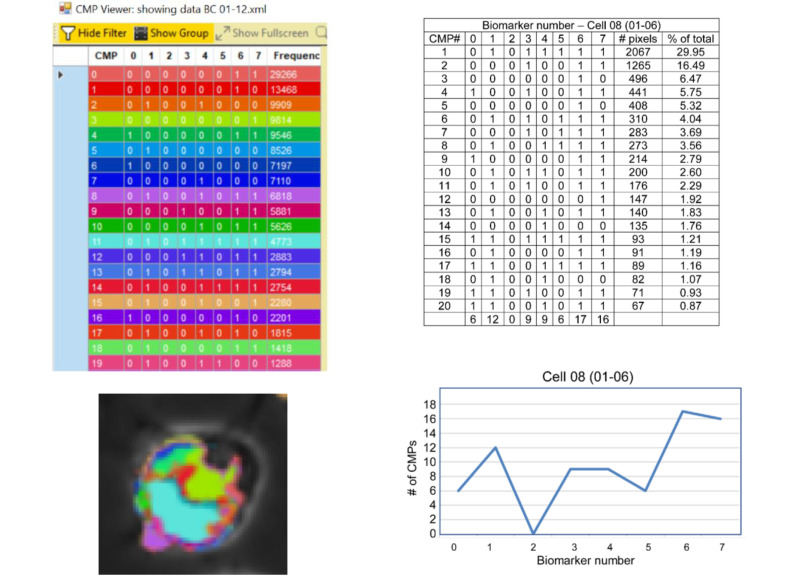

## Introduction

Macrophages are notoriously heterogeneous, especially after activation. The notion of macrophages being an M1 (inflammatory) or M2 (antiinflammatory) phenotype has evolved to models describing a spectrum of activation (1–6), with multiple distinct phenotypes depending on the activating stimulus or disease state. What is not well investigated is whether the “resting” phenotype is based on a single phenotype or a variety of phenotypes. Previous studies (7), including one employing the technology used here (8), showed that alveolar macrophages (AMs) in the “resting” or unstimulated state are heterogeneous and that various insults or stimulatory protocols may result in heterogeneous cell populations.

Surfactant protein A (SP-A) has surfactant-related functions and plays roles in host defense, many relating to innate immunity and involving AMs (7, 9–25). The responsible mechanisms are complex, especially in humans, where there are 2 functional SP-A genes (*Sftpa1* and *Sftpa2*) and their corresponding proteins (SP-A1 and SP-A2). Although SP-A1 and SP-A2 are similar, functional differences have been demonstrated. These studies were facilitated by expression of these proteins in vitro (11), and creation of humanized transgenic (hTG) mice on the SP-A–KO background, each carrying and expressing either SP-A1 or SP-A2 (12). SP-A1 and SP-A2 differentially affect AMs’ functions, including phagocytosis, cytokine production (11, 13–17), actin polymerization (7), and regulation of the proteome (18, 19), miRNome (20, 21), and gene expression (22). The significance of these differences is underscored in a pneumonia model by changes in lung function (23) and survival (24).

The AM proteome in SP-A–KO mice rescued with exogenous SP-A undergoes significant changes that vary with sex (19, 25). Treating SP-A–KO mice acutely with SP-A variants produces more pronounced effects (19) than those seen in hTG mice with chronic or constitutive exposure to SP-A (18). Building on previous studies, we investigated the effects of rescue with a single dose of exogenous SP-A1 on the AM toponome by investigating combinatorial molecular phenotypes (CMPs) of proteins (8) to study their spatial network within intact cells. CMP is a designation describing the presence or absence of all markers in a given pixel, rather than simply variations in expression of a single marker. An extensive AM heterogeneity/diversity was observed, with SP-A increasing AM phenotypic diversity, and CMP-based phenotypes were identified that were dependent on the presence or absence of SP-A. This study has a major advantage, working with intact, individual single cells, and avoiding blending (by disruption or homogenization) of diverse cell populations into a common pool.

We employed the Toponome Imaging System (TIS), also known as Imaging Cycler Microscopy, or Multi-epitope ligand cartography to study the expression and/or presence of multiple markers in their spatial networks in intact, individual AMs exposed chronically to different SP-A variants. With other technologies, including proteomics, the cells are disrupted, so data represent an average of all cells, rather than defining individual cells or groups of similar cells. TIS is a robotically controlled microscopic system developed by Schubert (26–30) that involves reiterative cycles of immunostaining, imaging, and photobleaching of FITC-conjugated antibodies. An important facet of TIS is that it uses dedicated software programs to process the resulting images to obtain a pixel-by-pixel map of the cells and show colocalization of proteins. The significance of this approach is that proteins rarely function in isolation and function often depends on proteins in their proximity as members of multiprotein complexes. TIS provides this information by mapping each cell for multiple markers. Thus, TIS does not simply colocalize proteins but allows identification and enumeration of supramolecular structures formed by protein clusters, termed CMPs, and identifies candidate proteins potentially involved in protein-protein interactions. In the composite images generated by TIS, there are 2*^n^* possible CMPs or marker combinations where *n* = the number of markers used. CMPs are used to characterize cells, enabling identification of cell subpopulations. There is evidence that various conditions cause differences in both the number and composition of CMPs, and some conditions may be identified by CMPs with a unique composition (29–31). TIS is an ideal tool for addressing AM heterogeneity and the spectrum of polarization occurring after activation (1–6).

Here, we studied CMP-based phenotypes that are unique or overlap among the 3 AM groups (KO, SP-A1, SP-A2) to gain insight into protein spatial network differences among these groups potentially underlying the observed functional differences at the baseline or “resting” state. We analyzed both all AMs within the captured images from each sample and individual cells in the same samples. The goal was to understand the effect of constitutively expressed SP-A on supramolecular protein structures or CMPs, use these to determine AM phenotypes in different mouse strains, and identify AM subgroups within the same strain. These CMP-based AM phenotypes may translate into functional differences as shown previously (26). Existing strategies and additional procedures developed here allowed us to characterize individual cells, compare cells from replicate samples, and compare and contrast individual cells from different groups. The data showed extensive CMP heterogeneity with no 2 cells being identical, but with CMP-based phenotypes found predominantly in one group or another, as well as overlap of CMPs among groups.

## Results

The results presented are from AMs from 3 mouse strains. To study the impact of constitutive exposure to SP-A1 or SP-A2, or the absence of SP-A (KO) on CMP-based AM phenotypes, we first analyzed whole images containing multiple cells, then individual AMs selected at random from each image. AMs were studied to distinguish AM CMP-based phenotypes among strains and CMP-based subgroups of phenotypes within each strain. Additional procedures were developed and 12 samples (4 samples/group) were studied for both, the whole-image and the individual cell analysis. For the latter, 168 cells (KO, *n* = 48; SP-A1, *n* = 57; SP-A2, *n* = 63) were studied.

### Whole-image analysis

#### Markers.

Markers used were selected from a larger collection of antibodies included in each TIS run. Fluorescent debris, bubbles, or other artifacts prevented us from using an image from 1 or more samples and resulted in the omission of that marker from the final image collection because we required artifact-free, high-quality images for all 12 samples. The 8 markers chosen (Table 1) produced the most reliable artifact-free signals in all 12 samples and allowed us to compare replicate samples from the same group and across groups. One biomarker was autofluorescence (AF) of the AMs (biomarker 0) at the beginning of the TIS run. AF varies from cell to cell and is a useful characteristic for analyzing myeloid cells (32). AF tends to be localized in intracytoplasmic organelles that may contain NAD(P)H, flavins, ceroid/lipofuscin, bilirubin, and porphyrins, among others (32), and is often punctate or granular in nature (8) (Supplemental Figure 1; supplemental material available online with this article; https://doi.org/10.1172/jci.insight.141410DS1). The positive organelles may be related to AM bactericidal capacity. AF was completely eliminated by the photobleaching cycles. Several markers (CD44, CD68, CD18) may play roles in phagocytosis, and many others are involved in cell-cell and cell-matrix interactions (CD44, CD68, CD40, CD45, CD18) or cell activation (CD44, CD68, CD40, CD45, CD18, TLR4, and Ly-6C).

#### Analysis of data from whole images.

Whole-image analysis included all AMs in the sample. This consisted of the visual field with a 63× objective and of 2048 × 2048 pixels. This study builds on our proteomics studies (9, 10), where we compared AMs from different hTG mice with SP-A–KO AMs. Here, we demonstrate changes in the AM toponome under similar conditions (18). The protocol for obtaining samples was identical to that used in proteomic studies (18) and uses many of the same analytical approaches employed previously (8). Whole-image analysis is comparable to studies (proteomic, gene expression, miRNome) where cell populations are disrupted before analysis, resulting in a mixing/averaging of diverse cell types. Here this averaging of cells was done not by physical cell disruption but rather by “digital homogenization.”

**Figure 1:**
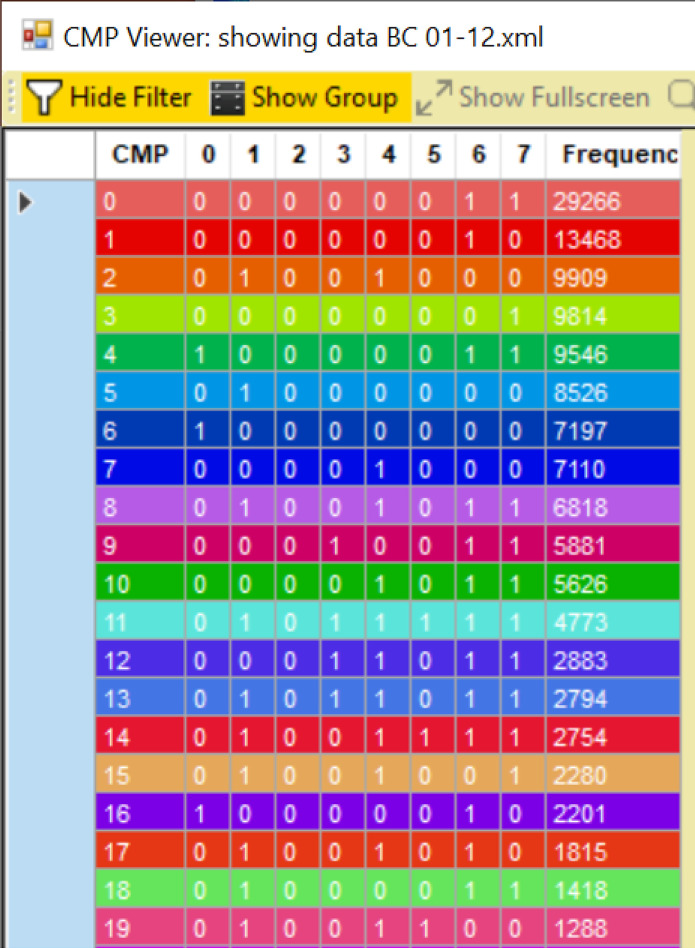
CMP chart. Screenshot of portion of chart generated by the MultiCompare program is shown. The chart is generated from an.xml file resulting from merging of all images for a given sample. CMPs are numbered (left-hand column beginning with 0) according to frequency (number of pixels; right-hand column). In each of 8 columns (numbered 0–7) the presence of a biomarker in a CMP is indicated by “1” or its absence by “0.” Colors are assigned automatically by the program. Identity of the biomarkers is given (Table 1).

**Figure 2:**
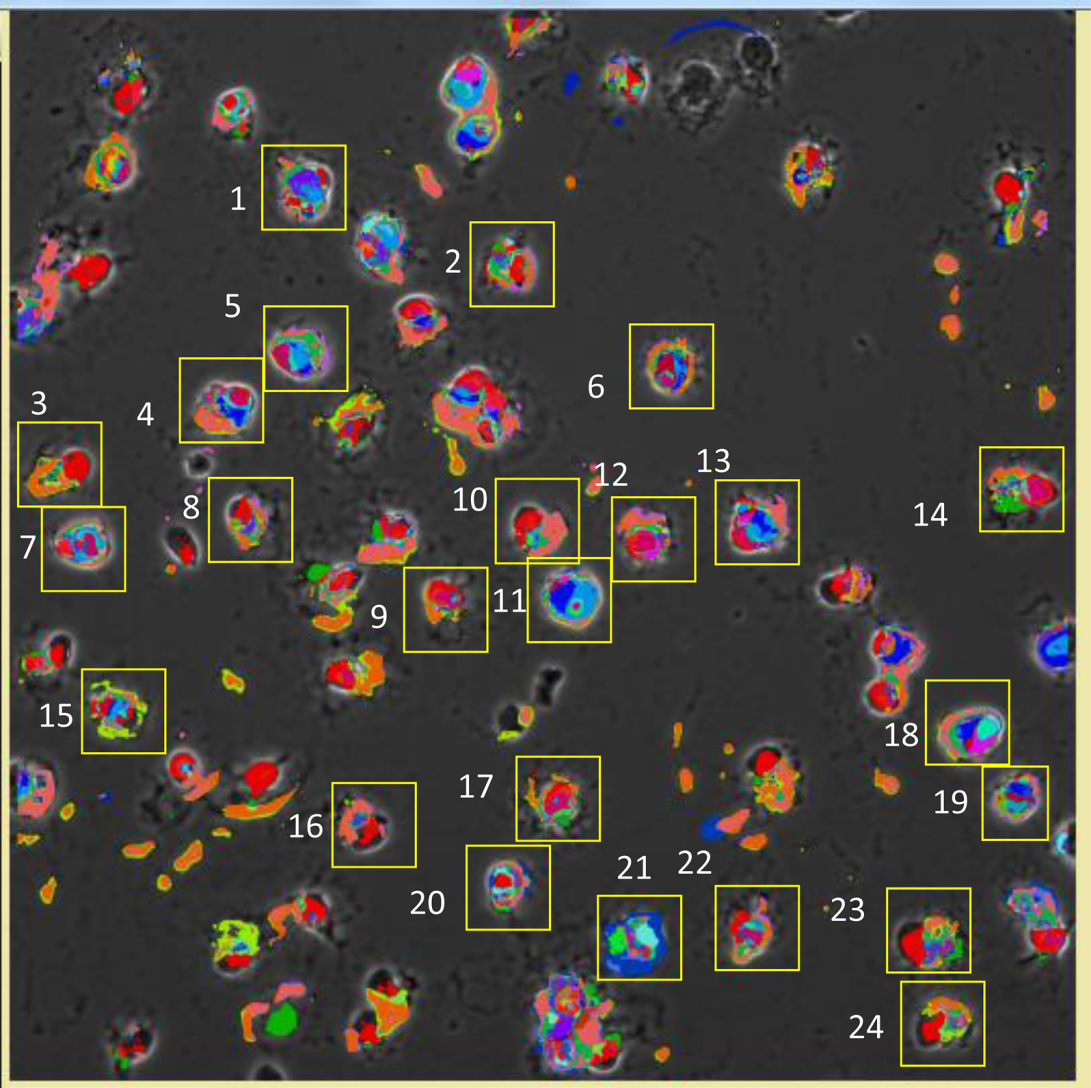
Pseudocolored image of cells. A representative image of 1 sample with pseudocolors assigned by the MultiCompare program and superimposed on the appropriate phase contrast image. Colors correspond to those for each CMP in the CMP chart (Figure 1). Note that cells or parts of cells that are not colored were areas that were below the threshold when binarization was done, rather than unstained during immunostaining. Numbered yellow squares indicate cells from that sample that were selected for individual analysis.

Image processing was as described (Supplemental Figure 2) (8). After binarization of all images from the full 63× field of view, CMP lists were generated with the MultiCompare program (Figure 1). A maximum of 2^8^ or 256 CMPs were possible with 8 markers. The 12 analyzed images had a range of CMPs from 69 to 187 (mean = 110 CMPs). Mean CMP numbers did not differ significantly (KO = 125; SP-A1 = 101; SP-A2 = 104). The number of cells in each image varied. Thus, frequencies (abundance; number of stained pixels) of the CMPs covered a wide range, so analysis of these images is qualitative rather than quantitative. The assigned colors (Figure 1) were also used for image pseudocoloring (Figure 2).

#### Biomarker intensity in whole images.

Levels of individual biomarkers in each group were assessed by intensity (i.e., estimated percentage), based on a zero-inflated Poisson’s regression model (33), of each biomarker in all positively stained pixels of each image and are listed (Table 2). For example, an intensity of 0.23 (marker 1 in KO) indicates that 23% of pixels in the 4 KO images contained that biomarker. Intensities of each biomarker are compared by calculating the ratios of each pair of estimated intensities. For example, an intensity ratio (for A/B) of 1 indicates that the estimated intensities of A and B were identical. The actual intensity ratios are not shown. The bottom part of Table 2 lists the adjusted *P* values, based on the false discovery rate (34), for the difference of that intensity ratio from a value of 1, indicating whether the intensity ratio of A to B differs significantly from identity. An adjusted *P* value of less than 0.05 was considered significant (bold type, Table 2). The values shown (Table 2) indicate, for example, that biomarkers 2 and 7 differed significantly in all comparisons, whereas biomarker 3 differed only between groups A and C. This indicates that biomarkers 2 and 7, but not 3, discriminate among all group comparisons.

#### Comparison of CMPs in whole images.

The similarity of samples (*n* = 4) in each group was assessed by comparing the 56 most abundant CMPs in each image as follows. We examined each of the 56 CMPs to determine whether they were also present in the top 56 CMPs of the 4 samples for each group. CMPs that were present in all 4 individuals of each group were tabulated as 4-of-4 (Table 3). These CMPs were considered “shared” among the 4 samples, and the data were viewed as a rough measure of similarity or conservation among samples. The SP-A1 group had the least similarity (or greatest heterogeneity), with only 9 of the 56 CMPs being shared among the 4 SP-A1 samples. The greatest degree of similarity (least heterogeneity) was in SP-A2, with 21 (of 56) CMPs shared by all 4 samples. The KO group was intermediate, with 16 of the 56 CMPs present in all samples. If we extended our analysis to include CMPs present in 3-of-4 samples, the same trend was present. Combining these 2 analyses, of the top 56 CMPs, 31, 20, or 38 CMPs were shared or present in all (4-of-4) or 3-of-4 AM samples from KO, SP-A1, or SP-A2 mice, respectively. Means of 4-of-4 + 3-of-4 totals from the groups (A, B, C) were compared by Kruskal-Wallis test and differed significantly (*P* = 0.007).

Also of interest in our compilation of 4-of-4 or 3-of-4 occurrences are the “less conserved” CMPs that were present in only 2-of-4 or 1-of-4 of samples in a given group. The presence of these CMPs in only 1 or 2 samples out of 4 may indicate that the regulation of these CMPs and the regulation of the markers comprising them are less stringent, at least with respect to the presence or absence of SP-A1 or SP-A2. Looking at mean values of 2-of-4 and 1-of-4 columns, and their totals in the right-hand column (Table 3), the SP-A1 group was more heterogeneous than either KO or SP-A2. The mean values of 2-of-4 + 1-of-4 totals were compared with a Kruskal-Wallis test and differed significantly (*P* = 0.007). As shown previously (8), AMs are heterogeneous, and while conditions in the alveolus, such as SP-A levels, may favor certain phenotypes, it is likely that most phenotypes are found in more than one group, although their relative abundance may vary.

Next, we studied whether the shared/conserved CMPs (4-of-4, 3-of-4) were present across all groups (KO, SP-A1, SP-A2) by assessing their presence in either all 4 or 3 members of each group. The underlying hypothesis for this type of analysis was that shared CMPs or CMPs being conserved in all 3 groups (KO, SP-A1, SP-A2) were not regulated (either positively or negatively) by SP-A1 or SP-A2. For example, the 9 CMPs present in all 4 samples (4-of-4) from the SP-A1 group (Table 3) were also present in 4-of-4 or 3-of-4 members of the other 2 groups (KO, SP-A2), indicating that their expression was independent of the SP-A status. In contrast to SP-A1 4-of-4 CMPs, we found that 4 out of 11 3-of-4 SP-A1 CMPs were present in only a single sample (1-of-4) from either the KO or SP-A2, indicating perhaps that for optimal expression of these 4 3-of-4 CMPs, SP-A1 was necessary. Although some conserved CMPs in KO AMs were present in both SP-A1 and SP-A2 AMs, indicating a lack of SP-A dependency, we found some of the KO CMPs to be absent in either both, or one, of the SP-A groups. So in cases where CMPs in KO AMs were present in SP-A1 (but not in SP-A2), we can postulate that the SP-A2 presence has an inhibitory effect on the expression of those CMPs. We also found a few CMPs that were absent in KO (or present in only a single sample) but present in 4-of-4 or 3-of-4 samples from either SP-A1 or SP-A2. This likely indicates that the expression of those CMPs is dependent on the presence of either SP-A1 or SP-A2. These comparisons provide valuable insight into the complexity of molecular phenotypes present in AMs from different mouse strains, and the differences and similarities of CMPs among groups. In addition, they provide a basis to speculate about the regulation/interaction of some markers in the form of CMPs, although they do not prove that a potential regulatory influence occurs.

### Single-cell analysis

To gain insight into AM phenotype and diversity defined by CMPs, we investigated single cells. Unlike the whole-image analysis where all imaged cells in each sample, in effect, underwent a digital homogenization, the single-cell analysis characterized each cell individually. The selection criteria are described in Methods.

#### CMP and pixel analysis of individual cells.

We obtained coordinates of single cells within each sample and mapped pixel addresses of 168 cells from 12 samples comprising 3 groups. The coordinates for each cell were used to compile a list of CMPs in that cell as described (8).

The total number of CMP-containing pixels for each cell ranged from 2415 to 16,066 pixels and comprised nearly 1.1 million pixels for all selected cells. This is the number of pixels in each cell containing a positive signal for at least 1 marker, not the area of each cell. There are often areas within the cells that are below the binarization threshold for positivity or unstained, and these areas are not included in pixel totals. The estimated geometric means of positively stained areas for each group and their confidence limits (Table 4) and pairwise comparison of these means (Table 5) are shown. SP-A1 (group B) AMs had the highest geometric mean and SP-A2 (group C) the lowest. In pairwise comparisons between groups, all differed significantly (*P* < 0.0001). As stated above, these values represent the positively stained pixels, and not necessarily cell size, and the significance of differences remains to be determined.

A method developed previously (8) was used to obtain a graphic representation of the CMP content of each cell. The 20 most abundant CMPs in each cell were analyzed. If there were slightly fewer than 20 CMPs in a given cell, all CMPs for that cell were used. For most of the cells the top 20 CMPs comprised more than 95% of the total pixels for that cell. The mean values for the percentage coverage by the top 20 CMPs were 96.85%, 97.78%, and 97.98% for groups A, B, C, respectively, so restricting the analysis to the top 20 did not exclude much data. This is not to say that the less abundant CMPs may not be important, but these are beyond the scope of this study.

**Figure 3:**
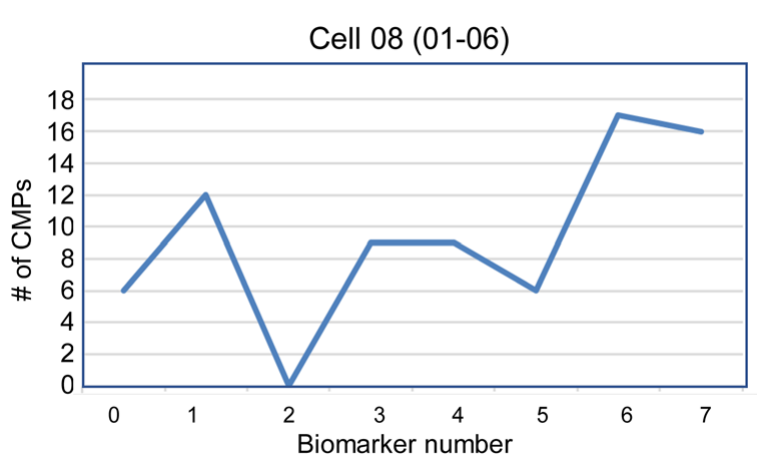
Cell signatures. For each of the 20 most abundant CMPs (Table 6), the total number of CMPs containing a given marker is shown at the bottom of Table 6 and was used to produce the line graph shown. For example, the line graph shows that marker 0 is found in 6 CMPs and marker 2 in 0 CMPs. The line graph provides a graphical summary of the most abundant biomarkers for each cell. The identity of the biomarkers is given (Table 1).

In the 20 most abundant CMPs for each cell, presence (designated 1) or absence (designated 0) of each biomarker in that CMP was tabulated, and data from a representative cell (cell 08 from 01-06 sample) are shown (Table 6). The total number of CMPs containing a given biomarker is shown at the bottom of the table. For example, in this cell, 6 CMPs contain marker 0 and 12 contain marker 1. A line graph with the values (i.e., total CMPs from Table 6) is shown (Figure 3). These graphs provided a distinct signature for each cell. It is worth noting that from the 168-cell data set, no 2 cells were identical. This high level of diversity/heterogeneity confirmed previous observations (8). Here we showed this was also the case in AMs from mouse strains constitutively expressing SP-A1 or SP-A2.

#### Cluster analysis of each experimental group.

**Figure 4:**
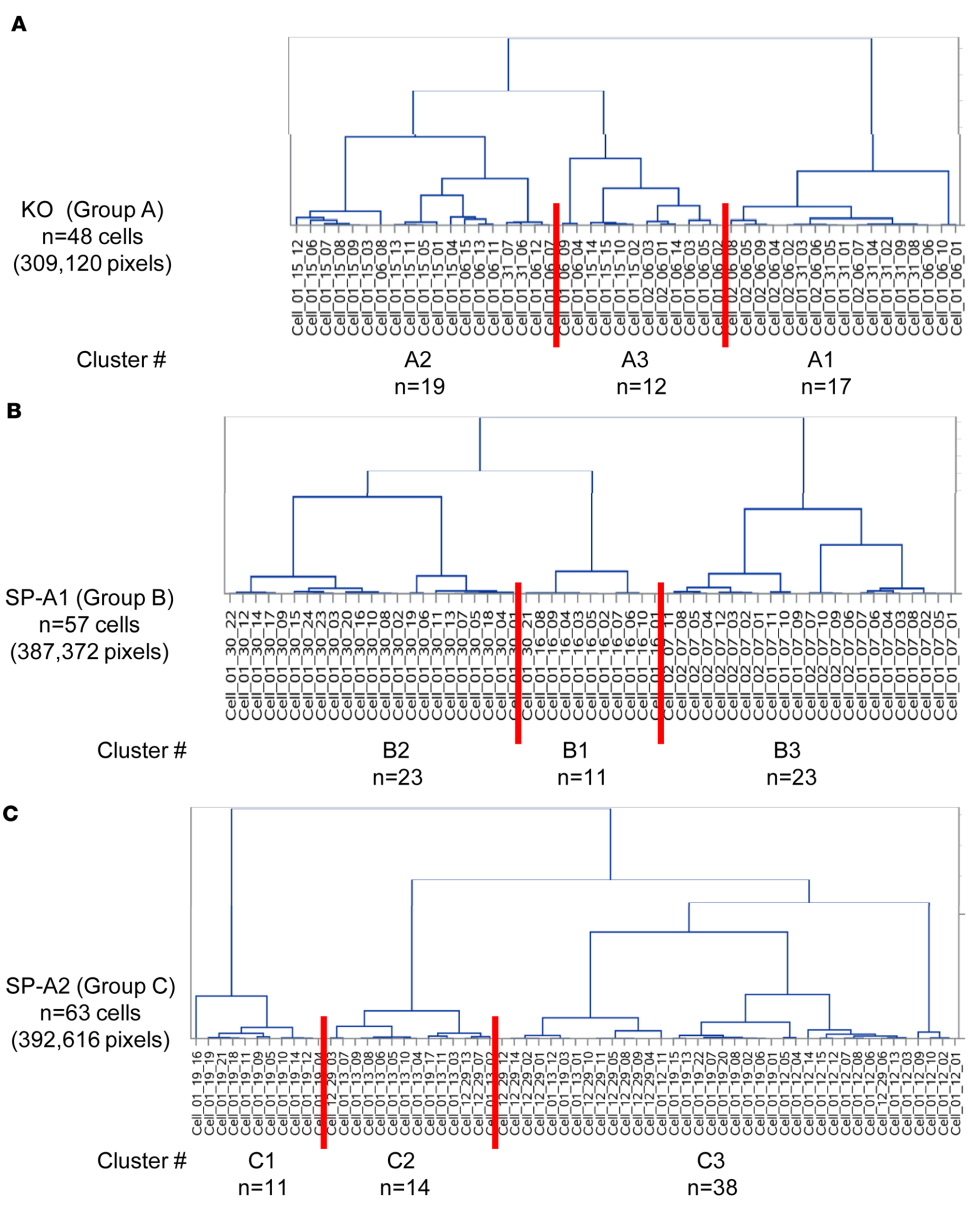
Group-wise clustering analysis. Clustering was performed on the cells of each experimental group separately: group A (**A**), group B (**B**), and group C (**C**). The identity of each group (KO, SP-A1, SP-A2), the number of cells/group, and the number of pixels are shown on the left of each panel. The dendrogram is shown on the right with the list of included cells across the bottom. The 3 main clusters from each group are delineated by red lines drawn through the cell lists. The designation for each cluster (i.e., A1, A2, A3) and the number of cells/cluster are given.

**Figure 5:**
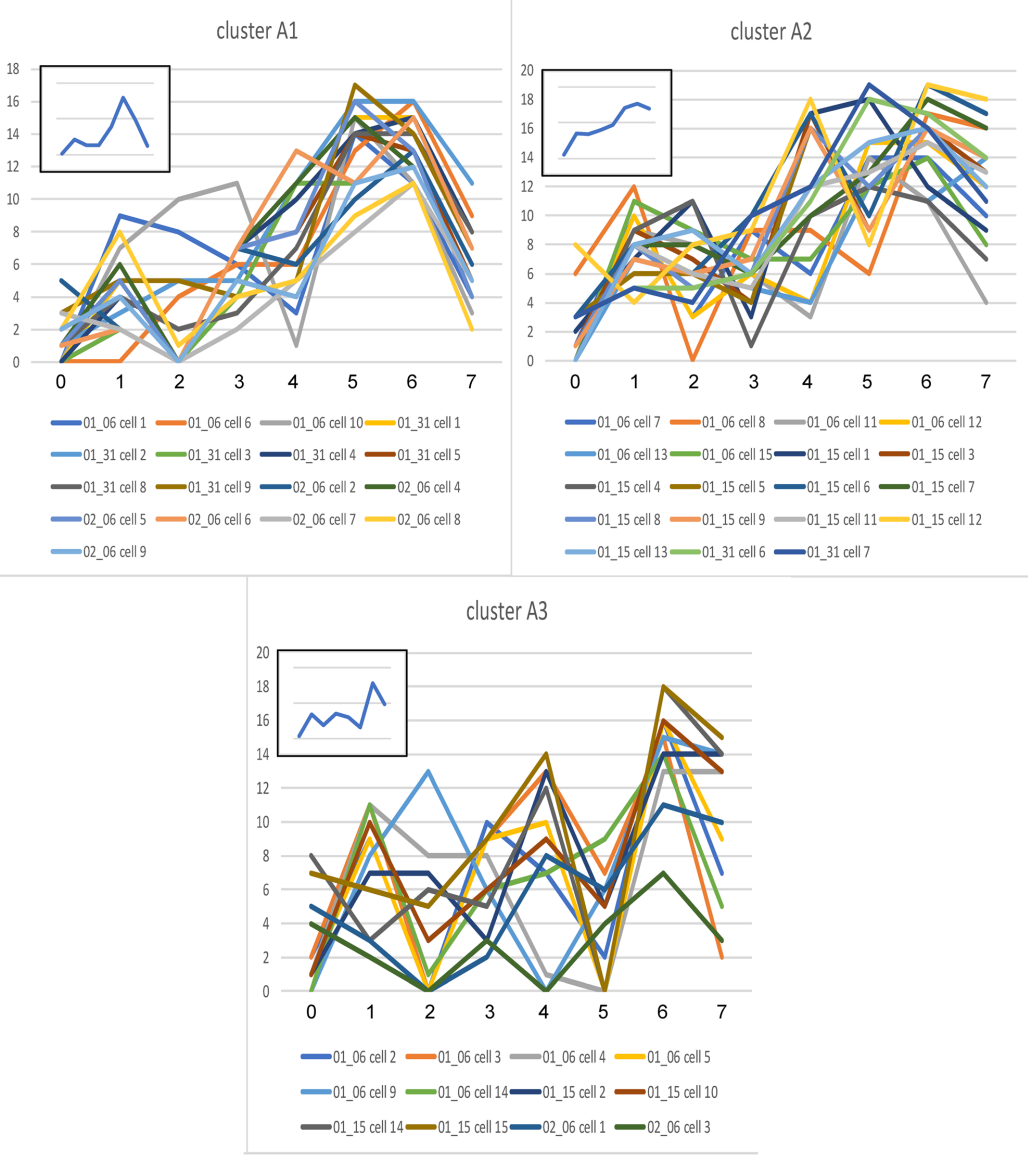
Signatures for cells in KO clusters. The line graphs from the cells composing each of the 3 main clusters (A1, A2, A3 — Figure 4A) for all AMs from KO mice are shown. The *x* axis (numbered 0–7) shows individual biomarkers. The identity of the biomarkers is given (Table 1). The *y* axis shows the number of CMPs in the 20 most abundant CMPs containing each biomarker. Individual cells composing each cluster are provided below each set of graphs. The graph in the inset serves as a summary graph for the multiple individual graphs shown in the main panel and shows the average percentage or intensity (% of pixels containing each biomarker/total # of pixels occupied by CMPs) for each cell in the cluster containing each biomarker.

**Figure 6:**
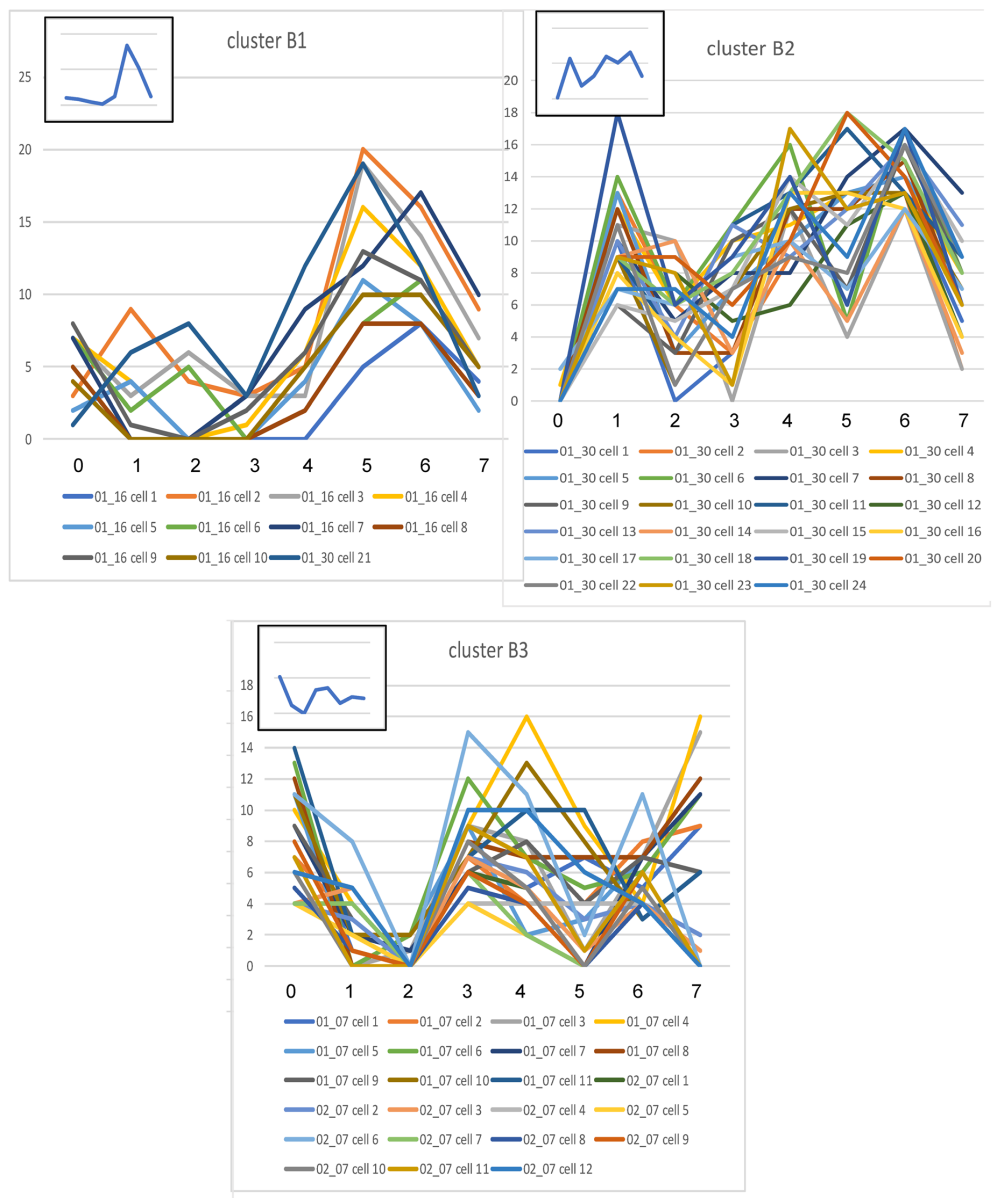
Signatures for cells in SP-A1 clusters. Line graphs from the cells composing each of the 3 main clusters (B1, B2, B3 — Figure 4B) for all AMs from SP-A1 mice are shown. Details are as described in legend for Figure 5.

**Figure 7:**
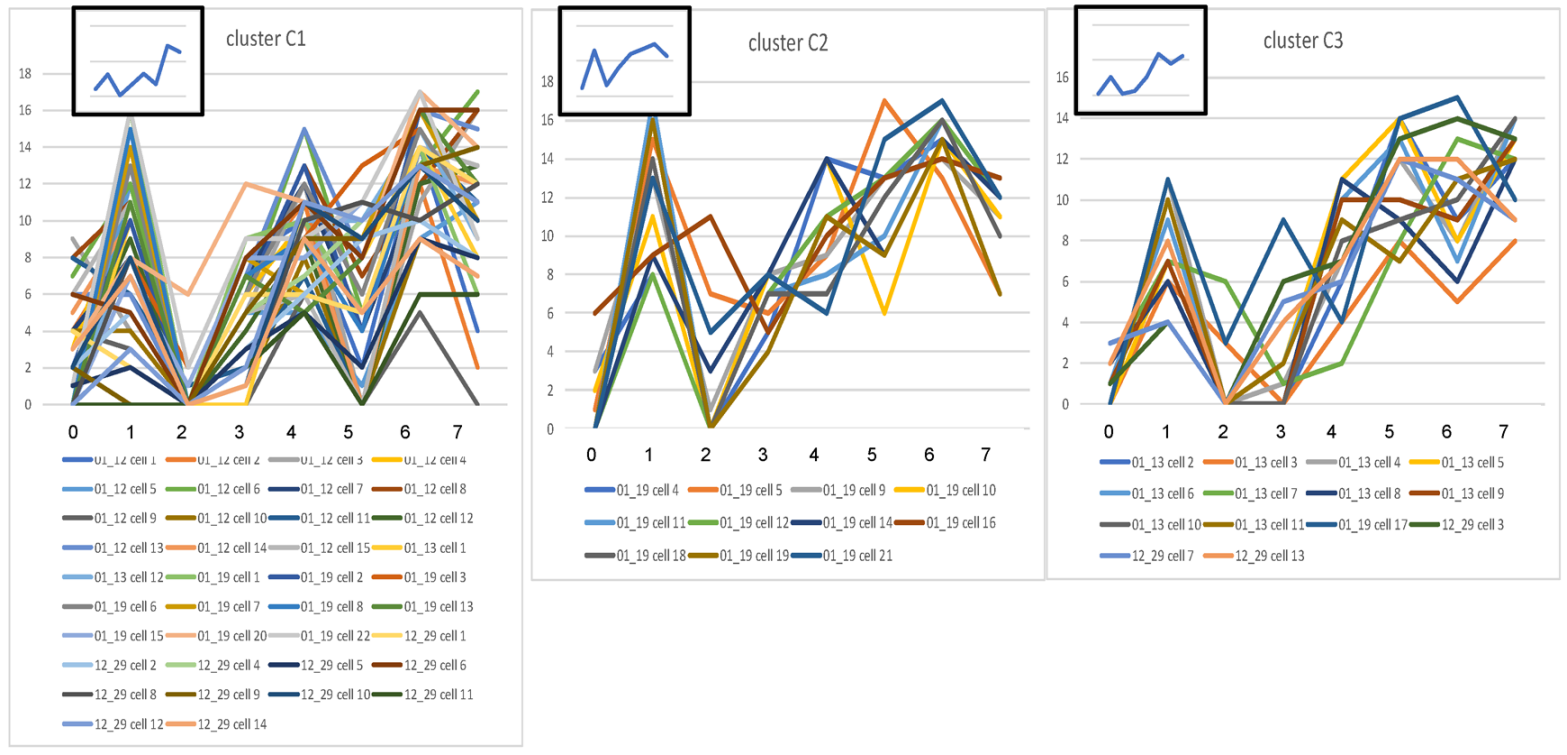
Signatures for cells in SP-A2 clusters. Line graphs from the cells composing each of the 3 main clusters (C1, C2, C3 — Figure 4C) for all AMs from SP-A2 mice are shown. Details are as described in legend for Figure 5.

The data of each group were subjected to cluster analysis to determine subgroups with shared similarities in their CMPs. The data were comprised of positively stained pixels from each cell and its biomarker content. This revealed that each group (KO, SP-A1, SP-A2) was divided into 3 main clusters (Figure 4, A–C). The graphical signatures for all cells in each cluster are shown (Figure 5, Figure 6, and Figure 7). When all line graphs/signatures (such as Figure 3) for all cells in a given cluster were plotted, a characteristic shape was discernible for that cluster. In some cases the graphs for clusters from different groups were remarkably similar (Figure 5, cluster A1; Figure 6, cluster B1). In other cases, although some features were similar, other parts were more variable. For example (Figure 5, cluster A3 and Figure 6, cluster B2), the profiles for biomarkers 6 and 7 are similar, but other markers are quite different. As part of the cluster analysis, tables were generated to show the mean percentage of each biomarker in the pixels that were included in the cells of each cluster. These values were plotted as line graphs (insets in Figure 5, Figure 6, and Figure 7) depicting a summary graph for each cluster. Each line graph (inset) was almost identical to the shape resulting from plotting all the signatures for individual cells for that cluster (i.e., compare the shape of lines of a given cluster with the summary line in the inset).

It should also be noted that graphs generated for the 3 clusters from each group were in agreement with data presented (Table 3), which showed the similarity of samples within experimental groups. SP-A2 was the most similar, and SP-A1 was the most diverse. The 3 sets of line graphs for SP-A1 (Figure 6) show 3 markedly different patterns. Conversely, the 3 sets of line graphs for SP-A2 (Figure 7) are remarkably similar, particularly with respect to markers 0, 1, 2, 4, and 6.

#### Cluster analysis of all individual cells.

**Figure 8:**
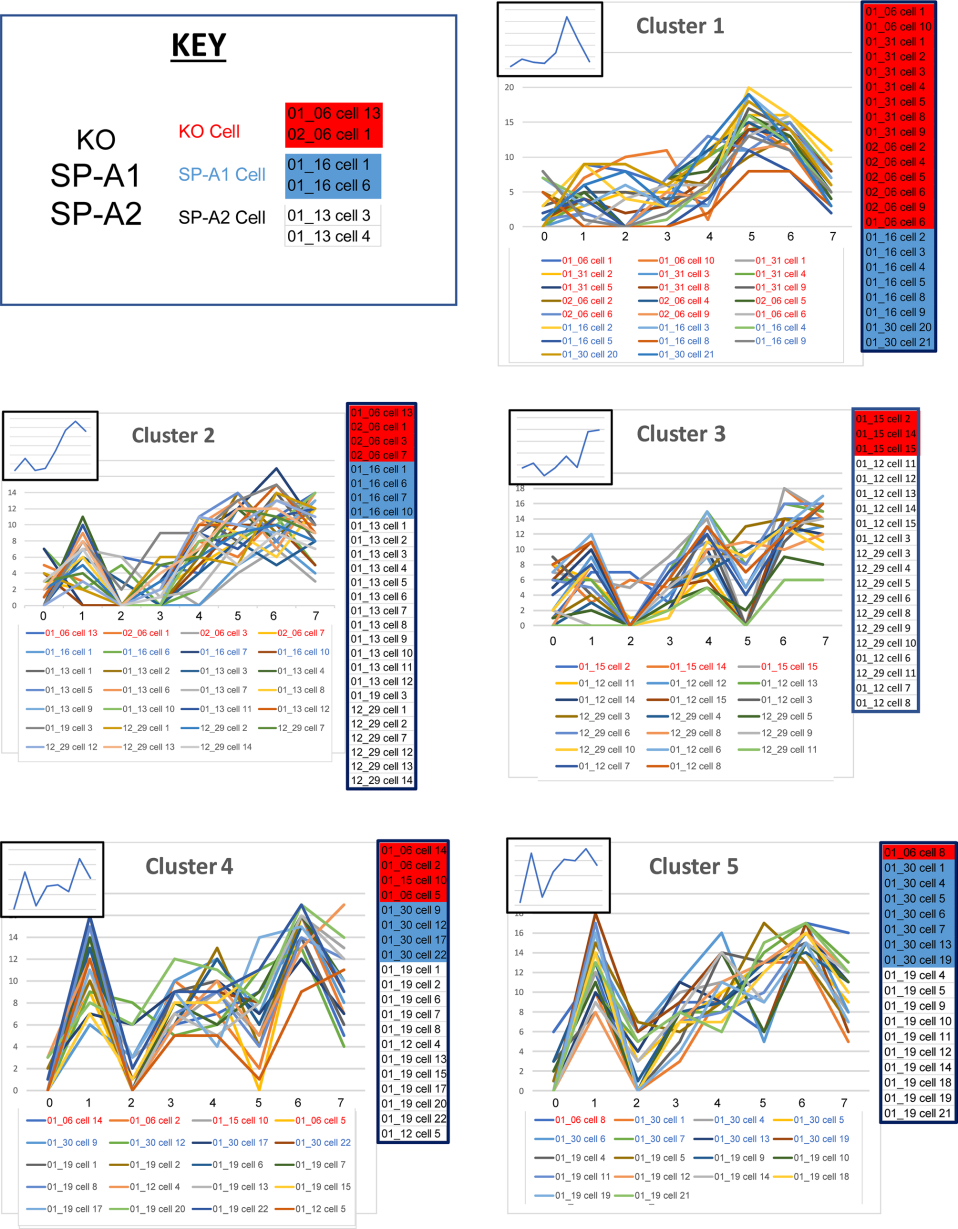
Profiles of clusters 1–5 (out of 10). For this analysis the full data set (all groups — KO, SP-A1, SP-A2) is used, and a cluster analysis is shown (Supplemental Figure 3). For clusters 1–5 defined by the dendrogram, the line graphs (signatures) for included cells are shown, along with insets showing the graph generated by the mean biomarker percentages (Table 7). Clusters 6–10 are in Figure 9. The IDs of cells in each cluster are below the graphs (red, KO; blue, SP-A1; black, SP-A2), and a color-coded list (red, KO; blue, SP-A1; white, SP-A2) of the included cells is presented on the right side of each graph to facilitate comparison.

**Figure 9:**
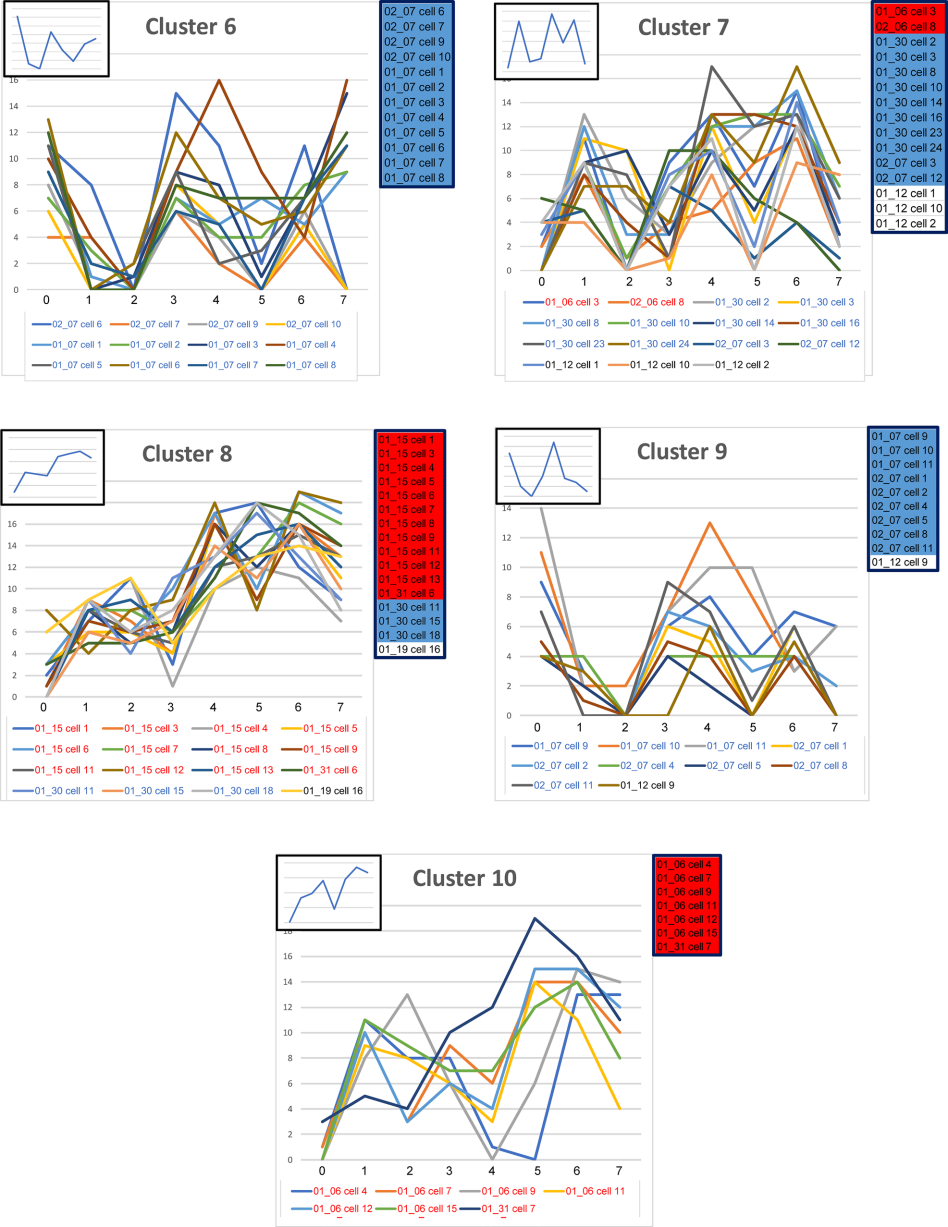
Profiles for clusters 6–10 (out of 10). Graphs are shown for clusters 6–10. See legend for Figure 8 for details.

A cluster analysis using 168 cells from KO, SP-A1, and SP-A2 was conducted, generating a dendrogram that defined 10 clusters. The dendrogram for this analysis (Supplemental Figure 3) and the line graphs for all cells in each of the 10 clusters (without distinction whether the cells are KO, SP-A1, or SP-A2), plus the summary insets, are shown for each cluster (Figure 8 and Figure 9). The actual values for the mean percentages or intensities for each biomarker in a given cluster are graphed in the insets and are listed (Table 7). Also, in each panel (Figure 8 and Figure 9), a color-coded list of cell IDs is included for that cluster (red, KO; blue, SP-A1; white, SP-A2). Most clusters included cells from more than one group, although in many clusters a single group constituted the majority of cells. The number of cells from each group included in each of the 10 clusters is listed (Table 8).

The line graphs, particularly the insets, provide insight into the composition of the clusters and how they were delineated. For example, Figure 8, cluster 1, shows a graph with low levels of expression in markers 0, 1, 2, and 3, then a sharp rise and high levels of marker 5. Cluster 1 consists of KO and SP-A1 cells, and its pattern is similar to that seen in graphs when each experimental group was separately subjected to cluster analysis. In this case cluster 1 (Figure 8) is similar to cluster A1 (KO) (Figure 5) and to cluster B1 (SP-A1) (Figure 6), where each was the result of the specific experimental group-wise clustering analysis (Figure 5, Figure 6, and Figure 7). Therefore, cluster 1 (Figure 8) exhibits a phenotype shown in the specific group-wise analysis to be present in KO and SP-A1 cells but absent in SP-A2 cells. There is another example (Figure 8, cluster 3) where most cells are SP-A2 cells and a few are KO. The line graph when all cells are considered together (Figure 8, cluster 3) is similar to cluster C3 (Figure 7) and cluster A3 (Figure 5), where SP-A2 and KO cells were analyzed separately, respectively. Together, the cluster analysis of each experimental group (Figure 5, Figure 6, and Figure 7) and/or of all groups combined (Figure 8) show that the different clusters/phenotypes exhibit distinct CMP-based characteristics and demonstrate that these CMP-based phenotypes are usually not exclusive but may exhibit some overlap among groups. Moreover, the presence or absence of SP-A and the type of SP-A (SP-A1 or SP-A2) is important in determining the phenotype of AMs. There were 2 clusters in the 10-cluster dendrogram that were exclusive to a given group. These were clusters 6 and 10 (Figure 9). Cluster 6 was from SP-A1 and cluster 10 from KO. However, none of the exclusive phenotypes were from SP-A2 AMs.

#### Definition of more exclusive phenotypes with additional selection criteria.

**Figure 10:**
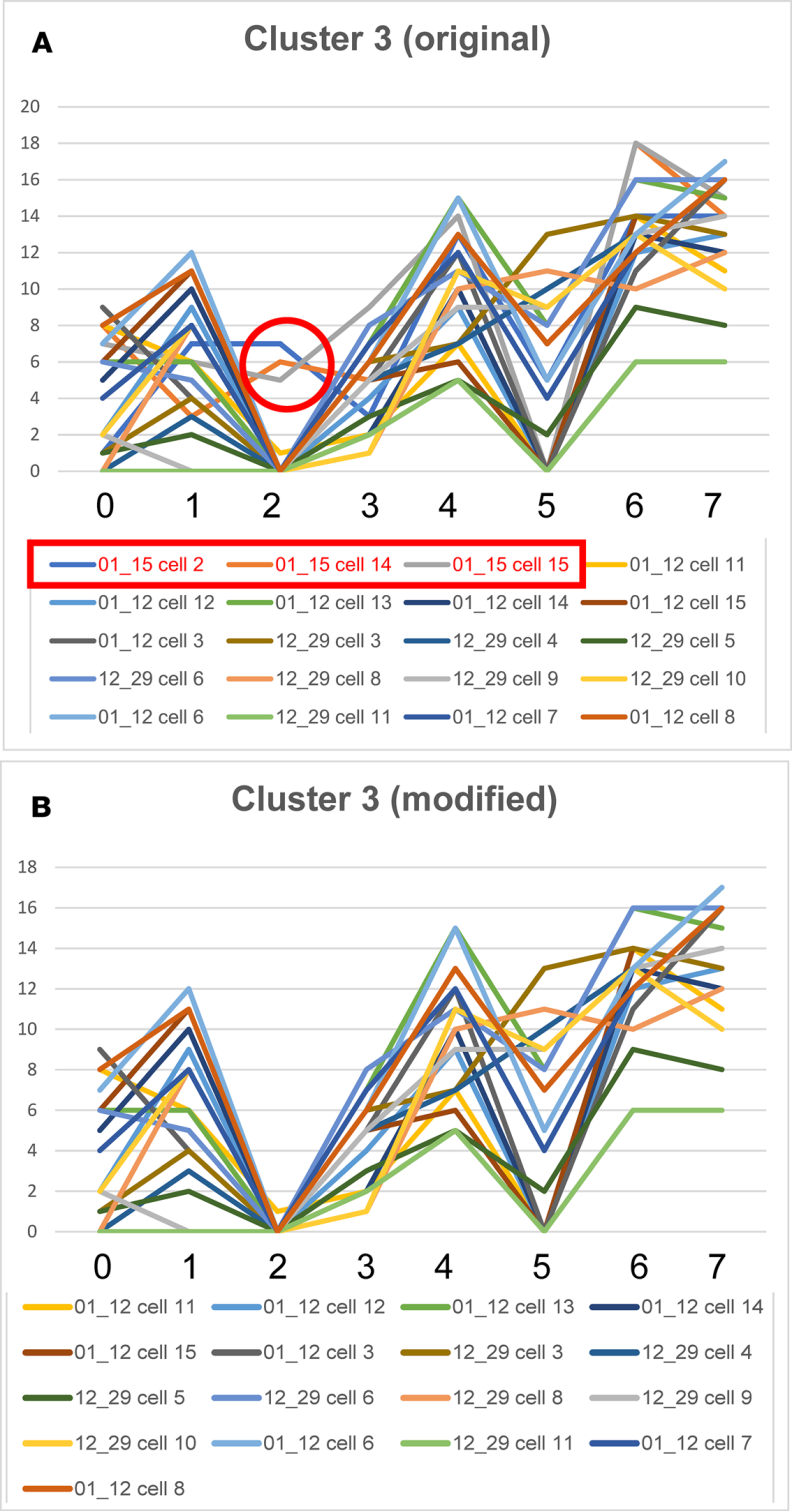
Refining selections. Cells included in cluster 3 (from Figure 8) and their graphs are shown (**A**). All cells are SP-A2, except for the first 3 in the legend of panel **A** (shown in the red rectangle). The red circle encloses a portion of the graph/signature for these 3 KO cells. When these 3 KO cells are removed from the graph (**B**), the resulting subset is composed entirely of SP-A2 AMs. The identity of the biomarkers is given (Table 1).

For the remaining 7 clusters (Figure 8 and Figure 9), additional criteria defined phenotypic groups. For example, Figure 10A shows the graphic signatures for cluster 3 (Figure 8; Supplemental Figure 3) and the cell IDs for the 20 cells from which the graphic signature is derived (shown below the graphs). Of the 20 cells, 3 (shown in red and enclosed in the red rectangle) are part of KO (group A). Coincidentally, in these 3 cells, biomarker 2 exhibited values greater than 4, and this portion of their line graphs is enclosed by the red circle. The other 17 cells, however, are from SP-A2 (group C). When we eliminate the 3 KO cells with values for biomarker 2>3, the resulting graphs (Figure 10B) are all similar to one another in terms of inflections and deflections in the graphs, and all consist of cells from SP-A2 AMs. This type of strategy can be employed to probe a set of similar cells and refine that set to generate a new set of AMs with a greater degree of CMP-based phenotypic similarity to one another.

A strategy described previously (8) was also used to identify cell subgroups that were almost exclusive for each group, although some overlap with other groups was common. First, for each biomarker, the range of its presence in all cells of the data set was determined in order to define high and low levels of that marker (Table 9). For example, a biomarker present in all 20 of the most abundant CMPs in at least 1 cell might have a range of 0–20 (i.e., biomarker 5), although other biomarkers may have a smaller range (i.e., biomarker 0; range 0–14). We took the midpoint of this range to define the “high limit” for each biomarker. In the first example (range 0–20), the presence of a biomarker in ≥10 CMPs would constitute a high level, and if the marker was present in <10 CMPs, that was considered low (≤9 = low). In the second example (range 0–14), the presence in ≥7 CMPs constitutes a high level and <7 a low level. The resulting subset of cells was then probed to determine whether there were other CMPs that discriminate between groups. Table 9 provides a guide for biomarkers that might be useful for discriminating among groups.

**Figure 11:**
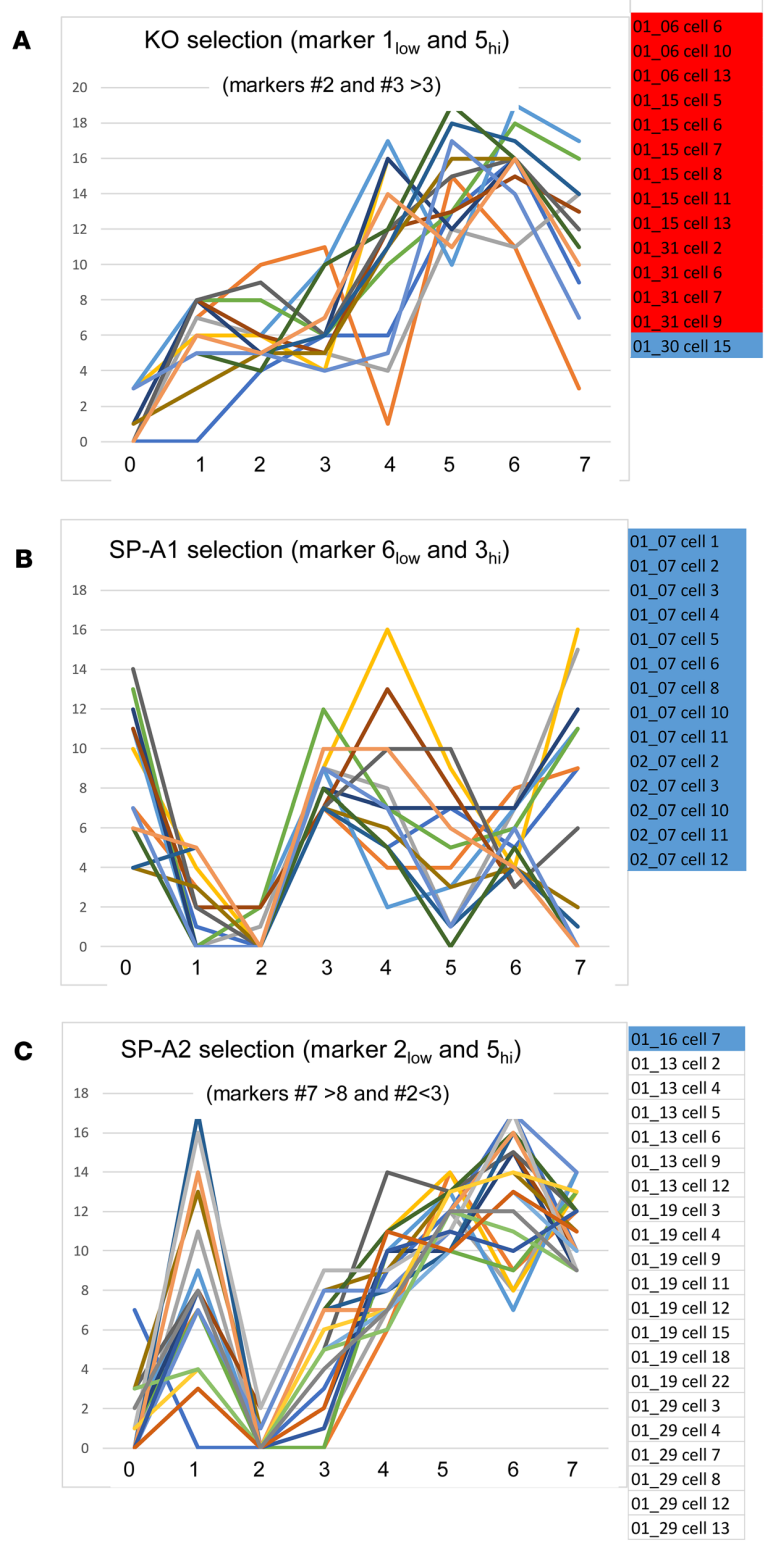
Selection criteria to define exclusive (or highly enriched) phenotypes. The lists of included cells (on right) are color-coded as in Figure 8. Examples are shown for enriched selections of KO cells (**A**), SP-A1 cells (**B**), and SP-A2 cells (**C**). In each graph, the title indicates the main selection criteria (i.e., low levels of biomarker 1 and high levels of biomarker 5 in panel **A**). Other selection criteria used are shown in smaller type under the main panel titles (i.e., biomarkers 2 and 3>3 in panel **A**). The identity of the biomarkers is given (Table 1).

Cells were selected (Figure 11A) with low levels (<9) of biomarker 1 and high levels of biomarker 5 (≥10). With these criteria 48 cells were selected from the data set, but when the group was restricted to those that had levels of biomarkers 2>3 and 3>3, a subgroup of 14 cells was obtained, 13 of which were in KO (group A). Cells (Figure 11B) with low levels of biomarker 6 (<8) and high levels of marker 3 (≥7) were selected (Table 9). This selection resulted in a group composed entirely of 14 AMs from SP-A1. In Figure 11C cells were initially selected with low levels of biomarker 2 (<6) and high levels of biomarker 5 (≥10), and the selection was further refined by restricting it to cells with high levels of marker 7 (≥9), then by further limiting biomarker 2 to low levels (<3). The resulting subgroup consisted of 21 cells, 20 of which were from SP-A2 (group C). In the 3 cases above, it was possible to generate subset of cells whose graphs had similar characteristics in terms of high and low levels of some of the biomarkers. These types of CMP-based selections provide a means for finding subgroups of cells that are exclusive, or nearly exclusive, to a single group. As with other selection strategies, as more criteria are applied to the selection, specificity increases, potentially providing more uniform cell populations that may better define AM function.

## Discussion

The human surfactant proteins, SP-A1 and SP-A2, have a differential impact on AM function and gene expression, and AM function and gene expression profiles differ significantly from AMs derived from mice lacking SP-A. Our goal was to gain insight into patterns of protein expression at baseline or in the “resting” state to explain the observed functional and regulatory differences among AMs constitutively exposed to SP-A1 or SP-A2 or AMs that were never exposed to SP-A (KO). Toward this, we employed TIS, a technology permitting multiple proteins to be colocalized in intact single cells, potentially providing insight into multiprotein or supramolecular complexes or CMPs that are more likely to be the basis for cellular function than isolated individual proteins. We studied male mice because studies have demonstrated sex differences of SP-A effects on AMs (7, 19, 20, 22–25). We found: (a) AMs are heterogeneous as shown previously in KO and SP-A1–rescued KO mice (8), with no 2 cells being identical; (b) the different groups (KO, SP-A1, SP-A2) showed distinct differences in CMP-based phenotypes; and (c) CMP-based cellular phenotypes showed that SP-A1 AMs are more heterogeneous than either KO or SP-A2 AMs and are rarely exclusive to a single group, although they may be highly enriched in one group versus another. Our results offer a new, slightly different perspective on the increasingly popular view that macrophage activation gives rise to a spectrum of heterogeneous subsets, namely, that the diversity following activation is the result of multiple resting phenotypes.

The unique strength of TIS is that expression levels and molecular patterns consisting of multiple markers (termed CMPs) are considered simultaneously, rather than focusing on a single marker or several markers. The rationale for this approach is that most proteins function as a component of a supramolecular complex of several proteins that may work together. This is readily illustrated by numerous pathway diagrams that permeate the scientific literature, such as the LPS/CD14/TLR4/MyD88-mediated LPS receptor pathway (35).

The unit of measure in this study is the CMP, a constellation of different biomarkers (8 in this study) exhibiting a variety of functions found in different combinations of proteins composing molecular patterns representing different CMP-based phenotypes. The assumption throughout this work is that AMs with similar CMPs have similar functional phenotypes. The CMP-based similarity/heterogeneity of images across the 3 experimental groups was assessed as described previously (8). Both the whole-image and individual cell data not only confirmed this heterogeneity but also showed it to be true in all mouse strains studied with TIS.

Whole-image and individual single-cell data differ significantly. The whole-image data are more similar to studies in which samples of AMs are considered as a whole (i.e., cells are homogenized), despite their acknowledged heterogeneity. The single-cell analysis provides a tool where intact, individual cells are characterized. However, in both cases (whole image and single cell), the analyses allowed us to compare replicate samples from each experimental group and to compare the 3 groups. Because TIS identifies molecular patterns of colocalized, potentially interacting proteins in intact cells that may mediate a given function, TIS provides a significant insight into AM molecular patterns that may underlie function. A CMP analysis is, in part, independent of whether the level of a given protein changes under different circumstances, but rather depends on the combinations of that protein with other potentially interacting proteins in its proximity.

### Whole-image analysis.

A CMP-based measure of heterogeneity/similarity showed that the 4 SP-A2 samples shared many of their most abundant CMPs, indicating a considerable degree of similarity among AMs from the 4 samples in this group. However, this was not the case for SP-A1 AM samples, where there were about half as many shared CMPs, compared with SP-A2, demonstrating far more diversity across the samples in SP-A1. Notably, the KO was intermediate between SP-A1 and SP-A2. Together, these observations are both interesting and puzzling, because in a number of AMs’ innate immune functions, such as bacterial phagocytosis (13–15) and proinflammatory cytokine production (11, 16, 17), SP-A2 exhibits higher activity, followed by SP-A1 and then KO. Moreover, a higher degree of AM binding has been shown by SP-A2 than SP-A1, with no difference in binding between SP-A1 and SP-A2 observed when a nonphagocytic cell line was used (24), indicating that macrophage-specific cell surface proteins are involved in the differential binding. Furthermore, when binding kinetics were investigated (36), SP-A2 exhibited a markedly higher binding capacity to AM compared with SP-A1. SP-A1, in contrast to SP-A2, showed no binding to AMs that were not previously exposed to SP-A (i.e., in KO AMs), underscoring potential complexities of SP-A interactions with AMs. In fact, when AMs chronically exposed to SP-A1 were used, the SP-A2 binding capacity was reduced, indicating that SP-A1 may have an inhibitory effect on AM binding.

Taken together, the present and published data of the impact of SP-A genotype on AM function and CMP diversity at first glance may be viewed as being puzzling and as having apparent discrepancies. The puzzling point is that SP-A1 showed more CMP diversity than the other 2 groups, although the magnitude of the SP-A1 effect on AM function or on a macrophage-like cell line is lower than that of SP-A2 (11, 13–17). In a study of the AM mRNome after ozone exposure, SP-A1 (unlike SP-A2) had no significant effect on the miRNome (20). However, the observation that SP-A1 has a stimulatory effect on AM function (albeit not at the same level as SP-A2) and an inhibitory effect on SP-A2 binding, or no significant effect as is the case with the AM miRNome, may help explain the CMP diversity observed in SP-A1 AMs. It is also possible that SP-A1 has a pronounced effect on AM functions and markers that have not yet been examined.

Of interest, the CMP-based observations (i.e., SP-A1 exhibiting higher diversity) share similarities with an AMs proteomics study where a greater number of proteins had their levels significantly changed in SP-A1 AMs compared with SP-A2 AMs (18). This was true whether the comparison for each was made versus KO or WT. Furthermore, the pattern of expression of various proteins in SP-A1 and SP-A2 in the proteomic study was largely in opposite directions (increase vs. decrease), although the expression of some proteins was in the same direction in both SP-A1 and SP-A2. Moreover, heatmaps based on various functional groups indicated that there were differences with regard to the functional phenotypes of SP-A1 and SP-A2. Although gel-based proteomics is a discovery technique with protein identification performed by mass spectrometry, whereas TIS targets specific proteins based on the availability of antibodies, the general observations made are consistent.

In aggregate, available data indicate that SP-A1 may be a general modulator of AM function and regulation and in the presence of SP-A2 may modify outcomes under certain circumstances. An example of this is seen where SP-A1 (although, as noted above, by itself did not affect significantly the AM miRNome) in the presence of SP-A2 under the same conditions enhanced the expression of pathways in AMs not present in SP-A2 alone (21). Thus, the higher CMP diversity in SP-A1 may be a way to accommodate multiple and apparently diverse modulatory functions on AMs. In contrast, SP-A2 and KO are shown to exhibit strong and poor innate host defense, respectively, and SP-A1 for the most part exhibited an intermediate level of activity. In a survival study after infection, SP-A2 mice had significantly better survival than SP-A1, and the SP-A1 survival was better than KO (24).

Furthermore, comparing shared CMPs among the different groups showed that a number of shared CMPs among the 4 samples in SP-A2 were only rarely found in KO or SP-A1 AMs, indicating a regulatory role for SP-A2 on these CMPs. A similar observation was made with some of the shared CMPs among the 4 samples in SP-A1 AMs, potentially implicating SP-A1 in their expression. However, there was a substantial overlap between groups, especially the KO and SP-A2. Notably, the majority of shared CMPs among the 4 samples in SP-A1 were also CMPs shared with the other groups (KO, SP-A2), indicating that these CMPs found in common in all 3 groups lack regulation of their constituents by the presence or absence of SP-A1 or SP-A2. The overlap and predominance of certain CMPs in one group or another may underlie and/or correlate with functional differences observed. For example, the AMs’ bacterial phagocytic index in the presence (or absence) of different SP-A variants is not an all-or-nothing effect but varies depending on the SP-A1 or SP-A2 variant. Thus, CMP-based phenotypes may underlie the degree and specificity of the SP-A1 and SP-A2 impact on AMs’ function and regulation.

### Individual cell analysis.

Although whole-image data provided some information of differing regulatory roles for SP-A1 and SP-A2, these differences become much clearer when individual cell data are studied. With TIS, we can effectively analyze single cells, compare them to one another and to cells from replicate samples, as well as compare CMP-based groups with distinct characteristics to identify phenotypes favored in the different mouse strains. The CMPs provided a means for categorizing heterogeneous cell populations and defining different phenotypes. The clustering analysis of the experimental groups showed that each group consisted of 3 major subgroups. The cell signature methodology, displaying a graphical representation of each of these subgroups, shows, as indicated by the whole-image data, overlap between groups, as well as CMP features unique to each group. These observations were confirmed by cluster analysis of the full complement of cells from all 3 groups, where overlap between groups was evident. However, in most cases a specific CMP-based phenotype was highly favored in one group versus another, and unique, group-specific features were observed. Moreover, analysis of individual single cells showed that more than half of SP-A1 AMs had either unique CMP phenotypes or phenotypes only rarely seen in KO and SP-A2 AMs, providing further support for the SP-A1 complexity.

AMs in this study were derived from untreated mice and reflect the steady state of AMs from lungs constitutively expressing SP-A1 or SP-A2. Although the results were consistent with our previous study (8) where KO mice were rescued with an acute exposure to exogenous SP-A1, the present study offers increased insight into the possible capabilities of AMs in lungs of these 3 mouse strains. In mice expressing SP-A1 or SP-A2 constitutively, differing kinetics of various components of the response is not a consideration. Also, although several biomarkers used are considered M1 markers (CD68, TLR4, Ly-6C), these AMs were not treated with agents that promote M1/M2 polarization (1, 3, 5), so effects of SP-A1 and SP-A2 on AM activation and polarization were not discussed. However, in all 3 strains at least 3 subclusters with distinct CMP-based phenotypes were found. Furthermore, the cluster analysis of all 168 cells revealed both strain specificity in some phenotypes and some overlap between strains. It is likely that these subclusters have functional differences and may represent subpopulations of cells with differing roles in lung host defense. This heterogeneity of resting AMs may contribute significantly to the extensive diversity found after activation (3).

In summary, the present data provide, for the first time to our knowledge, insight into molecular patterns that may underlie SP-A1– and SP-A2–mediated functional and regulatory differences of AMs, and these molecular patterns may be responsible for common, as well as unique, and/or additive/epistatic outcomes of AMs. The AM heterogeneity documented here by single-cell analysis provides insight into how this cell, via its interaction with the innate host defense molecules, SP-A1 and SP-A2, can perform a variety of functions to a varied degree, depending on the SP-A variant, and deal with many diverse challenges in the lung. It should be noted that AMs in these mice were exposed to comparable amounts of SP-A1 and SP-A2 (22). However, in humans the relative SP-A1 and SP-A2 levels in the bronchoalveolar lavage may vary drastically. In some cases the ratio of SP-A1 to total SP-A was shown to be significantly altered and this was associated with lung disease (37, 38). With this in mind, it is possible that CMP-based AM phenotypes under certain conditions may change or shift, adding further complexity into diverse AMs’ function and regulation.

## Methods

### Animals

Male mice of 3 strains (*n* = 4/strain) on the C57BL6/J–SP-A–KO background were used at 8–12 weeks. In addition to SP-A–KO, we employed 2 other strains, each containing a human SP-A1 or SP-A2 transgene (12). The SP-A1 and SP-A2 transgenic strains were developed and maintained by us at Penn State College of Medicine and are described (12). The SP-A–KO mice have been in our possession for more than 20 years and have been maintained, bred, and rederived by us. Mice were bred at the Penn State College of Medicine and raised under pathogen-free conditions or in barrier facilities with free access to food and water. There was no evidence of respiratory pathogens in sentinel animals housed in the same rooms. This study was approved by the Institutional Animal Care and Use Committee of the Penn State College of Medicine.

### Sample preparation

AMs were obtained and processed for TIS as described (8). Briefly, mice underwent bronchoalveolar lavage with phosphate-buffered saline (PBS) and 1 mM EDTA, and cells were isolated by centrifugation at 150*g* for 10 minutes at 4°C, washed, and counted. TIS slides were prepared by placing a 0.5-mm-thick plastic sheet with a circular opening (8 mm in diameter) onto a microscope slide. An aliquot (100 μL) of serum-free RPMI medium containing 100,000 cells was placed in the resulting well. Slides were placed in a CO_2_ incubator (45–60 minutes) to allow cells to adhere. AMs were gently washed by dipping the slides in PBS. Slides were air-dried (15 minutes), frozen, and stored at –80°C.

For TIS, slides were warmed to room temperature, and a 1.0-mm-thick rubber ring with a 10 mm diameter hole was placed over the cells. The cells were rehydrated, treated with normal goat serum diluted 1:50 with PBS for 1 hour, and washed repeatedly with PBS. The slide was placed on the microscope in the TIS chamber and a view field selected.

### Toponome Imaging System

TIS basic 4 (pi4 Robotics GmbH) was used (8). This consists of a climate-controlled cabinet containing a Zeiss AxioImager microscope with a Plan-Apochromat 63× water immersion objective, a digital imaging system (Finger Lakes Instrumentation, LLC), and a motorized pipette controlled by a robot. Software programs for data generation and analysis were developed by Reyk Hillert, Otto-von-Guericke-Universität Magdeburg, Magdeburg, Germany. The programs included Image Registrator v.1.1 (image alignment and background subtraction), Binary Center v.1.0.2 (binarization of images), MoPPi v.1.1.3.8 (conversion of binarized.png files into a single.xml file), and MultiCompare v.0.9.0 (extraction of CMP data from.xml files). TIS procedures and subsequent image analysis have been described (8) and are summarized (Supplemental Figure 2).

### Antibody calibration/optimization

Antibody calibration and optimization was done as described (8). All antibodies were conjugated with fluorescein isothiocyanate (FITC) and obtained commercially (Table 1). FITC was used because it can be photobleached after imaging and before immunostaining with additional antibodies. On AM samples similar to those we used for the study, each antibody was tested at different dilutions using an incubation time of 30 minutes to determine the concentration with the best signal-to-noise ratio. Exposure time for imaging was optimized to obtain good signals that were below saturation. After calibration and optimization, TIS runs were performed with the whole series of antibodies. After each round of imaging, bound FITC-conjugated reagents were photobleached and the next cycle was performed. Table 1 lists antibodies used, gene names (where appropriate), UniProt accession numbers, antibody source, and catalog number. Procedures for immunostaining and image analysis are outlined (Supplemental Figure 2) (8).

### Image processing for TIS

#### Whole-image analysis.

Images underwent initial processing with TIS software (Supplemental Figure 2) as described (8). Images were aligned to eliminate small shifts that may have occurred, ensuring that a given pixel is in the same position on all images. The shifted images underwent background subtraction. These steps used the Image Registrator program. Whole images contained 2048 × 2048 pixels, although a 15-pixel margin around the periphery of each image was not included. In our TIS system with a 63× objective, a pixel in the captured image covers an area of 117 nm × 117 nm.

The shifted, background-subtracted images for each marker were reviewed to ensure that they were free of artifacts and were subjected to binarization in the Binary Center program, where a positive signal was either present (designated 1) or absent (designated 0). Threshold setting for binarization of the images from each marker was done manually, and immunostained areas reaching the threshold were considered positive. All images used were processed for binarization on the same day to ensure consistency.

Using the MoPPi program, the binarized images for all 8 markers were merged into an.xml file listing every pixel and CMP present in that pixel. In this file each CMP is designated by an 8-character string of 1s (protein present) and 0s (protein absent) (i.e., 10101001).

The.xml files were imported into the MultiCompare program, generating a table of all CMPs. Each CMP was automatically assigned a color by the program, and its frequency (abundance) in the whole image was calculated. A representative sample of a portion of the resulting tables is shown (Figure 1). CMPs are numbered (left-hand column) in order of decreasing frequency (right-hand column), and the presence or absence of each biomarker (labeled 0 to 7) is indicated by 1s and 0s in the intervening columns. The frequency is the number of pixels in an image containing a particular CMP. Assigned colors are also used to superimpose pseudocoloring on a phase contrast image (Figure 2). Note that if the intensity of the immunofluorescence staining was below the threshold set during binarization, some cells or parts of cells were not pseudocolored. For additional analysis of CMPs, the.xml files for each sample were converted to text files and read into SAS, Version 9.4.

#### Analysis of whole images.

Whole images from KO, SP-A1, and SP-A2 were compared several ways. In one analysis we compared a data set in which we determined the number of identical or shared CMPs in all 4 (4-of-4) samples and 3 of 4 (3-of-4) samples for each group (Table 3). These 2 lists are added together in the next column as an index of how homogeneous or well conserved the abundant CMPs are within the group or how consistent each of the group members is with one another. Conversely, we considered the CMPs found in only 1 or 2 samples, and the final column where these values were summed from a group, to be an index of diversity or heterogeneity.

We then compared the groups with one another by taking each of the conserved or shared CMPs (i.e., 4-of-4 or 3-of-4) and determining whether it was present and conserved in the other groups. This comparison potentially provides information on whether a given CMP was group specific.

#### Single-cell analysis.

For single-cell analysis the pixel coordinates for selected cells were mapped with ImageJ software (NIH, https://imagej.nih.gov/ij/download.html) and converted to be compatible with the data in the SAS file of the whole-image data. Figure 2 shows an example of individual cells selected from an image. These coordinates were used to select the pixels comprising each cell and to determine the CMPs present in those pixels. This included only pixels positive for one or more markers. Pixels that were either unstained or below the threshold for positivity were excluded. SAS data sets were used to probe CMP data for single cells. This was done for a number of cells in each image. The selection criteria were that the selected single cells were grossly normal in appearance, did not touch or overlap another cell, and were not in the area within 15 pixels of the border of the image, which was not analyzed by the software. After determining the pixels occupied by each cell, the CMPs present in those pixels were determined by probing the SAS file containing the data for that image.

#### Cell signatures.

A table was generated for the 20 most abundant CMPs for each cell, or in rare cases where there were fewer than 20 CMPs, all the CMPs. The tables contain columns labeled 0–7 denoting each of the 8 markers (Table 6) and contain either a 0 or 1 depending on the absence or presence, respectively, of each biomarker. The bottom line of each table totals the number of CMPs in the top 20 containing each marker. Next, a line graph (Figure 3) was generated from each of the totals (bottom line of Table 6), providing a signature or snapshot of the makeup of each cell. The line graphs depict the total number of CMPs (out of the top 20 CMPs) containing each marker. These plots served as a summary of the marker content or CMP signature of the 20 top CMPs for each cell and allowed us to identify groups of cells with similar characteristics, even though their CMPs were not identical. Although Figure 3 represents a single cell, similar plots were made for the 168 cells comprising this study.

### Statistics

All statistical analyses were conducted using SAS, Version 9.4, after importing all pixel and CMP data. Ward’s minimum variance cluster analysis was applied, and dendrograms were generated to determine an appropriate number of clusters. Zero-inflated Poisson’s regression analyses (33) were applied to derive mean intensity values for each biomarker within each group, and intensity ratios of the biomarkers were constructed to compare the groups. Comparisons were conducted via 1-way ANOVA or Kruskal-Wallis test, and all *P* values were adjusted via the false discovery rate (34). *P* < 0.05 was considered significant. Line graphs for cell signatures were prepared in Microsoft Excel.

### Study approval

This study was approved by the Institutional Animal Care and Use Committee of the Penn State College of Medicine.

## Author contributions

DSP conducted TIS experiments, processed data, interpreted results, and wrote the manuscript. VMC converted.xml data files to SAS, allowing more extensive analysis; converted different pixel localization information into a common format; enabled individual cell data to be retrieved from whole-image data; and performed many of the statistical analyses on the data set. JW provided guidance and suggestions for conducting TIS experiments and processing data. LY was involved with data organization and analysis. DS provided helpful suggestions for antibody calibration, operation of the TIS system, and use of TIS software for processing of data. XZ purified exogenous SP-A, treated mice, recovered AMs from mice, and prepared slides for TIS. JF was responsible for overall direction of the project, including developing experimental design, interpreting results, and preparing the manuscript.

## Supplementary Material

Supplemental data

## Figures and Tables

**Table 9:**
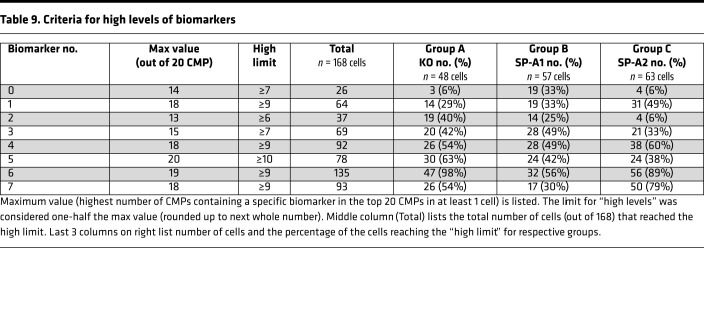
Criteria for high levels of biomarkers

**Table 8:**
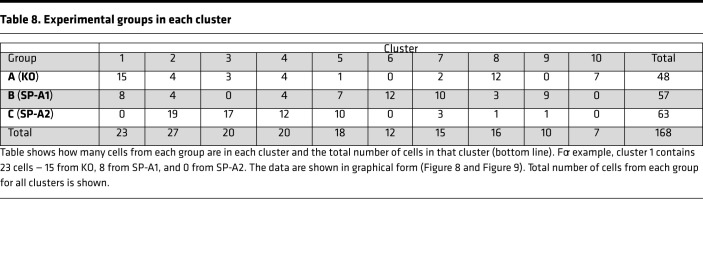
Experimental groups in each cluster

**Table 7:**
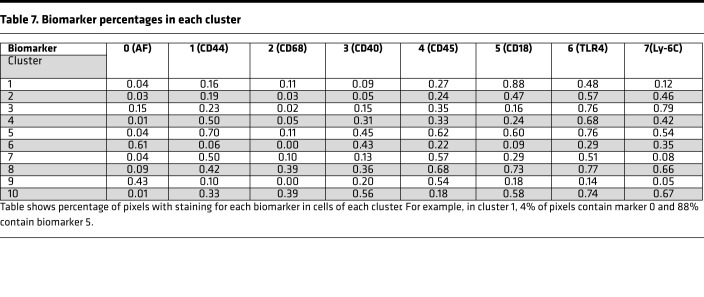
Biomarker percentages in each cluster

**Table 6:**
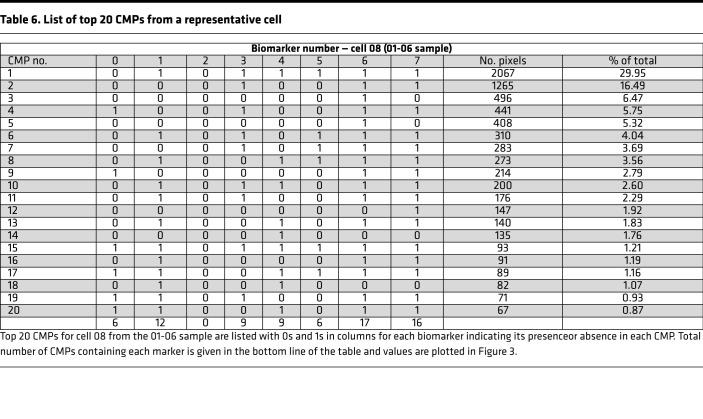
List of top 20 CMPs from a representative cell

**Table 5:**
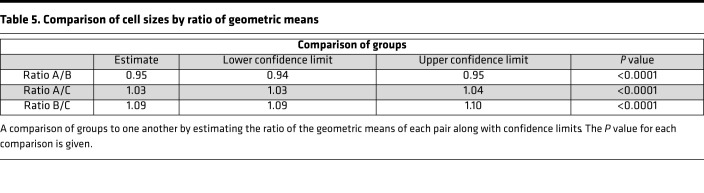
Comparison of cell sizes by ratio of geometric means

**Table 4:**
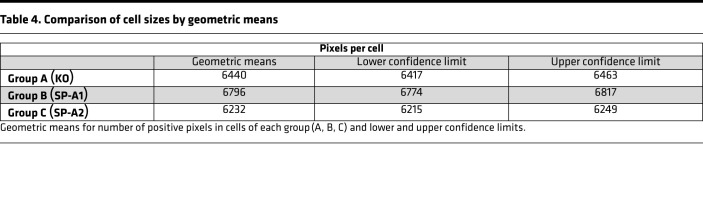
Comparison of cell sizes by geometric means

**Table 2:**
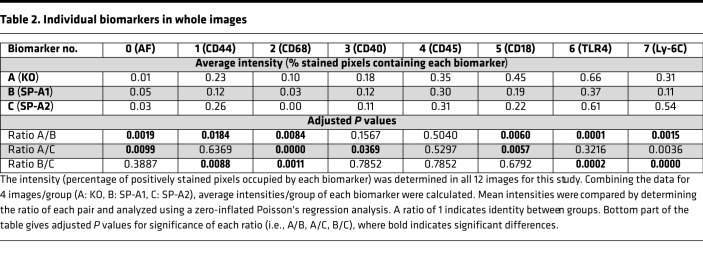
Individual biomarkers in whole images

**Table 3:**
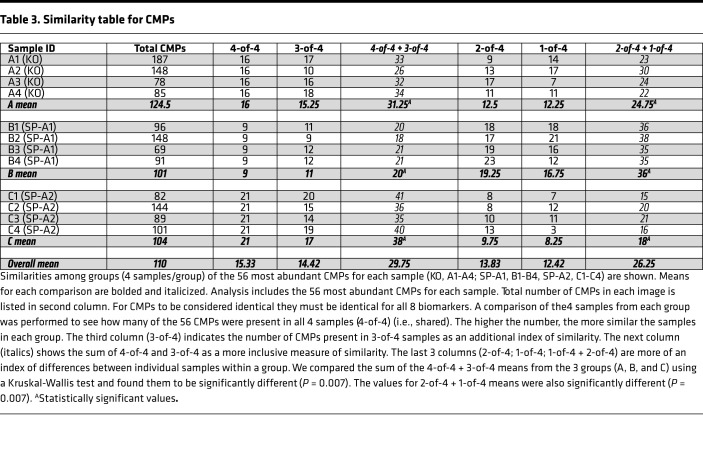
Similarity table for CMPs

**Table 1:**
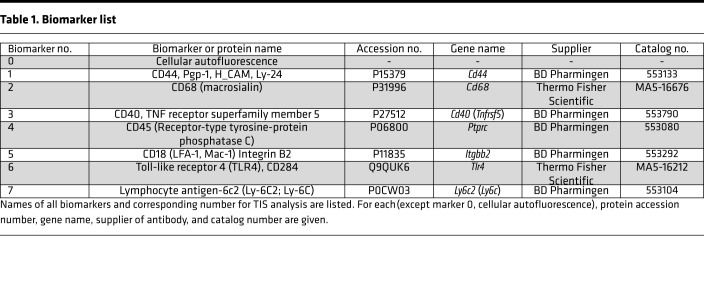
Biomarker list

## Version 1 11/03/2020

In-Press Preview

## Version 2 12/17/2020

Electronic publication
