# Supplementary material for: Differences in the alveolar macrophage toponome in humanized SP-A1 and SP-A2 transgenic mice
Source: JCI Insight. 2020 Dec 17;5(24):e141410. doi: 10.1172/jci.insight.141410 (PMC7819750; doi:10.1172/jci.insight.141410)

SUPPLEMENTARY FIGURES

Supplementary Figure 1. Autofluorescence of AM. A representative image shows the autofluorescence in a sample of AM. The inset shows an enlargement of a single cell.

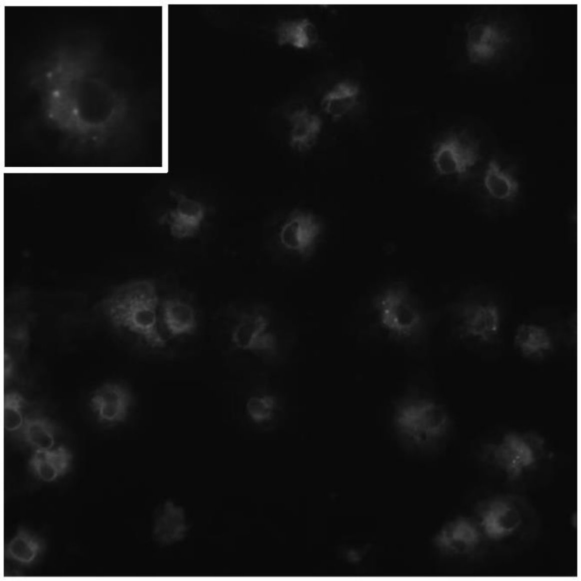

Supplementary Figure 2. Flow charts. Flow charts are shown for the TIS procedure and for Image Analysis.

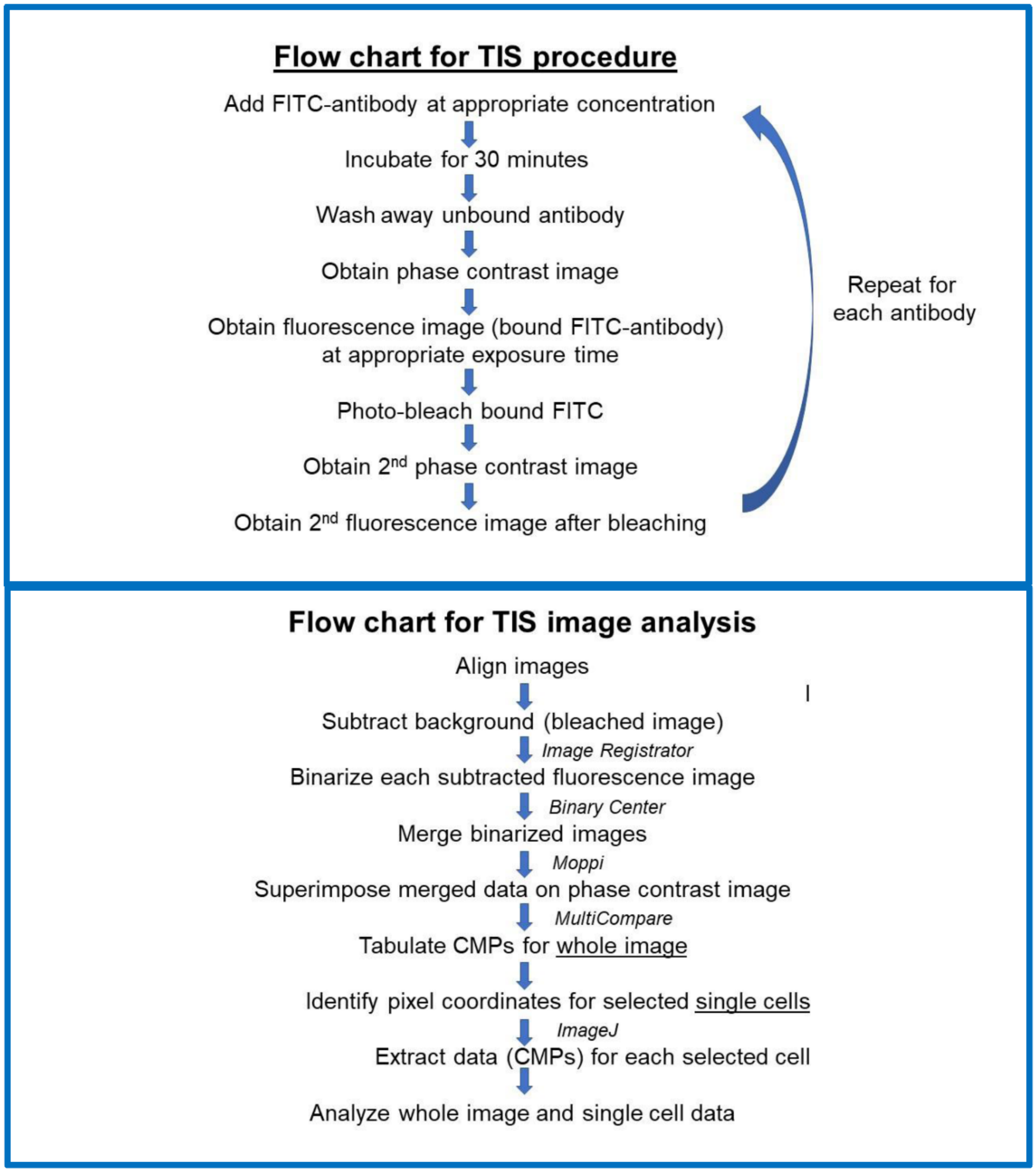

Supplementary Figure 3. Clustering analysis of all cells. Clustering analysis was performed on all cells (n=168 cells; 1,089,108 pixels) regardless of group (i.e. KO, SP-A1, SP-A2). Ten main clusters are indicated by vertical red lines. Cluster numbers (i.e. 1, 2, 3, etc), along with the number of cells in each cluster are shown. Details of each cluster are shown (Figures 8 and 9).

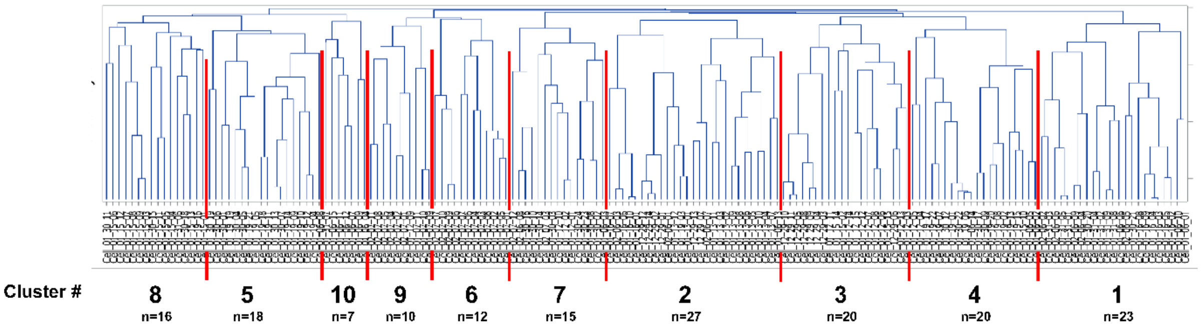

Supplement: Supplemental data [file jciinsight-5-141410-s030.pdf]
